# The International Skin Spectra Archive (ISSA): a multicultural human skin phenotype and colour spectra collection

**DOI:** 10.1038/s41597-025-04857-5

**Published:** 2025-03-23

**Authors:** Yan Lu, Kaida Xiao, Michael Pointer, Ruili He, Sicong Zhou, Ahmed Nasseraldin, Suchitra Sueeprasan, Cheng Gao, Changjun Li, Ali Sohaib, Yuanyuan He, Yoko Mizokami, Mengmeng Wang, Esther Perales Romero, Faraedon Zardawi, Lesley Gill, Ningfang Liao, Sophie Wuerger

**Affiliations:** 1 School of Design, University of Leeds, Leeds, UK; 2 School of Design, Dar Al-Hekma University, Jeddah, Saudi Arabia; 3 Faculty of Science, Chulalongkorn University, Bangkok, Thailand; 4 School of Computing, University of Science and Technology Liaoning, Anshan, China; 5 Nordson Corp, Dunstable, UK; 6 School of Engineering, Utsunomiya University, Utsunomiya, Japan; 7 Graduate School of Informatics, Chiba University, Chiba, Japan; 8 School of Digital Technology and Innovation Design, Jiangnan University, Wuxi, China; 9 Department of Optics, University of Alicante, Alicante, Spain; 10 School of Dentistry, University of Sulaimani, Sulaimani, Iraq; 11 School of Healthcare Science, Manchester Metropolitan University, Manchester, UK; 12 National Key Lab of Colour Science and Engineering, Beijing Institute of Technology, Beijing, China; 13 School of Psychology, University of Liverpool, Liverpool, UK

**Keywords:** Human behaviour, Tissues

## Abstract

This paper presents the International Skin Spectra Archive (ISSA), a multicultural human skin phenotype dataset, containing 15,256 records of both spectral and colorimetric data derived from 2,113 subjects. These measurements, collected between 2012 and 2024, come from eleven different datasets gathered by international laboratories across eight countries, all adhering to a uniform measurement protocol to ensure data consistency. The ISSA dataset addresses the inherent challenges in measuring human skin colour due to its complex structure and covers a wide variability in skin characteristics such as geography, ethnicity, age, gender, and body location. Providing a broad spectrum of human skin data, the ISSA dataset will advance our understanding of skin colour variations and their biological, cultural, and environmental influences. It will also serve as a crucial resource for scientific research and technological development across various fields where diverse and precise spectral and colour data of real human skin are essential.

## Background & Summary

Measuring the spectral properties of human skin and its variations has been fundamental to research in vision and evolution. As a multi-layered organ, human skin derives its colour mainly from the absorption characteristics of melanin in the epidermis and haemoglobin in the blood^1^. Melanin has adapted to balance protection from ultraviolet radiation with vitamin D synthesis, exhibiting colours from tan to white^2^. Haemoglobin, the red chromophore, introduces the distinctive ‘W’ shape in the skin reflectance spectrum^3^. This critical feature is hypothesised to influence the development of primate trichromacy for enhancing skin discrimination^4^. Skin colour is a highly variable human trait, with its evolution and genetic bases being complex and far from well-understood. Recent genetic studies have underscored the need for objective measurement data on diverse skin phenotypes to help understand this culturally and biologically important trait^5^.

The spectral composition of human skin determines its colour appearance perceived under various viewing conditions. Thus, reflectance measurements are crucial for assessing the real colour of human skin and reproducing its appearance across multiple fields, particularly in merging facial technologies. For instance, our familiarity with skin colour necessitates precise rendering and reproduction in digital imaging such as computer graphics, photography, cinematography, virtual reality (VR) and augmented reality (AR)^6–9^. Advances in 3D printing technology have highlighted the importance of accurate skin colour reproduction for creating skin prostheses^10,11^. In medical contexts, precise spectral data is vital for analysing skin chromophores to detect and diagnose various skin diseases and other health issues^12–15^. Such data also supports skin assessment, skincare product development, and preference identification in the cosmetics, personal healthcare, and medical beauty industries^16–19^. Furthermore, more inclusive skin datasets are necessary to enhance face recognition and biometric authentication technologies, particularly in data-driven artificial intelligence (AI) algorithms^20–23^.

While skin spectra data provide vital references for the aforementioned research and practical applications, achieving accurate measurements is challenging due to the uneven, multi-layered structure of the skin and the broad variability it exhibits. Measurement techniques and protocols, such as instrument type, aperture size, measurement distance, and pressure applied during measurement, can introduce considerable variation in results^24^. Additionally, the appearance of human skin varies widely affected by ethnicity, gender, age, body location, and even factors like diet, and health^19,25^. A comprehensive, comparable skin spectra dataset should account for the essential variations and adhere to a uniform protocol, with well-documented notes on measurement techniques and detailed subject information.

Previous efforts on collecting spectral datasets have focused on daylight and various natural objects, such as plants, with limited resources dedicated to human skin^26,27^. In 2003, the International Organisation for Standardization (ISO) released the standard ‘*Graphic technology - Standard object colour spectra database for colour reproduction evaluation (SOCS)’*, which included 8,213 human skin reflectance from six datasets: SHISEIDO, KAO, OOKA, KAWASAKI, OULU and SUN^28^. These reflectance data were primarily gathered from Japanese and Caucasian participants using different instrument types and measurement geometry, complicating comparisons between different datasets. Over the past two decades, several other skin datasets have been collected to meet varying research and industry needs^29–33^. However, none adequately represent demographic variations due to limited sample sizes or are easily integrated due to differing measurement methods. Furthermore, most of these datasets are not freely accessible to the public.

**Fig. 1:**
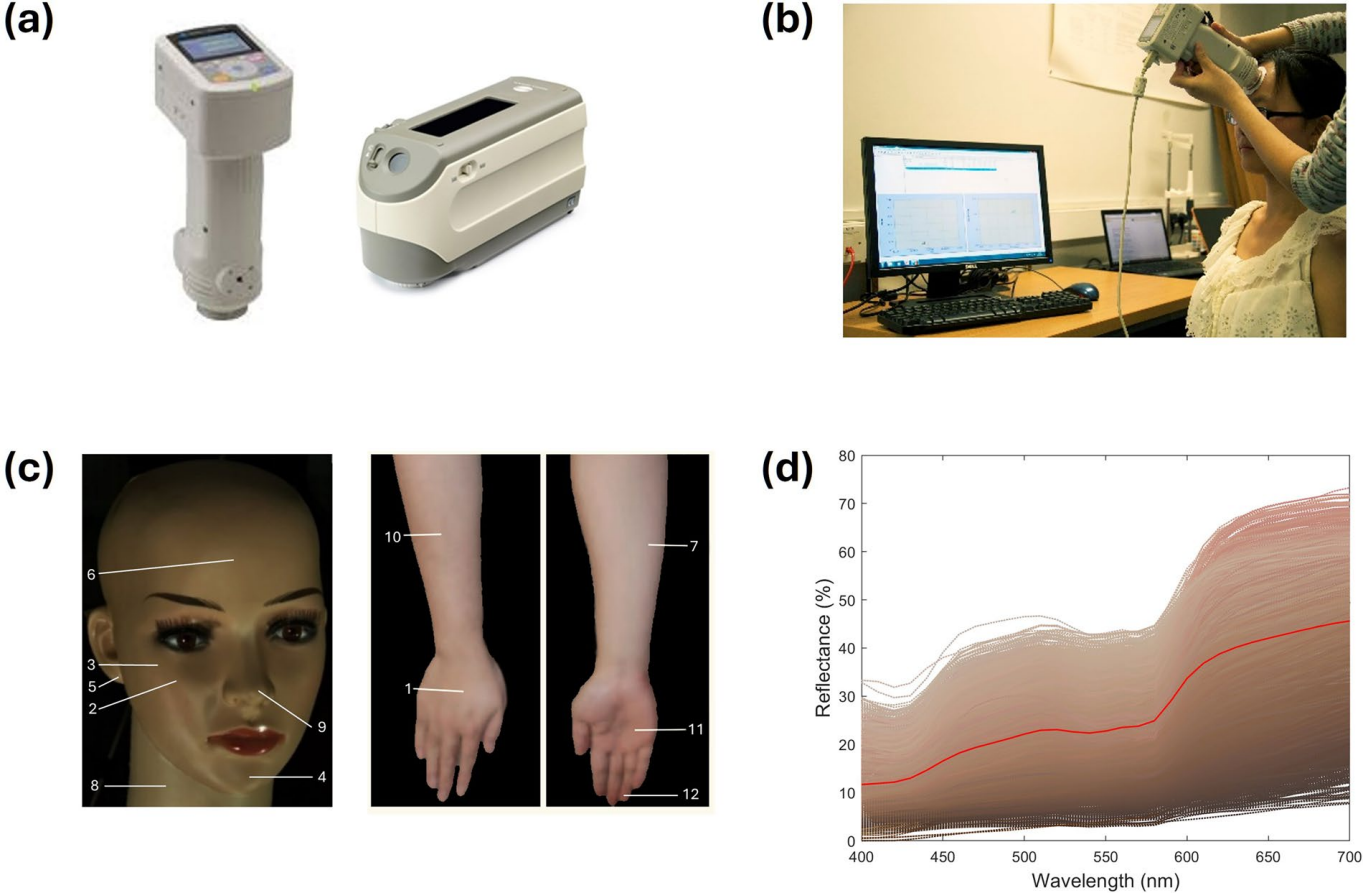
An overview of measurement tools and processes for skin spectra data collection (**a**) Measurement instruments displayed: on the left, the CM 700 d spectrophotometer; on the right, the CM 2600 d spectrophotometer. (**b**) The measurement process illustrated with a spectrophotometer positioned to measure the forehead of a subject. (**c**) Display of 12 standard body locations used for skin measurements. (**d**) Spectral reflectance across all skin measurements (N = 15,256) included in the dataset. Each dotted line represents the spectral reflectance from a specific body location of an individual subject, with the colour of each line providing a visual approximation of the skin colour; the solid red line indicates the average spectral reflectance of human skin.

In 2013, the International Commission on Illumination (CIE) initiated the Technical Committee TC 1–92, Measurement of Human Skin Colour, to investigate the measurement uncertainty and recommend protocols for good measurement practice. Guided by this committee, we collected eleven high-quality skin spectra datasets between 2012 and 2024 at thirteen global laboratories across eight countries: the UK, Spain, China, Japan, Pakistan, Thailand, Iraq, and Saudi Arabia. This effort led to the establishment of a new spectra dataset for human skin - the International Skin Spectra Archive (ISSA). The purpose of the dataset was to provide a publicly accessible resource of diverse human skin spectra data, supporting both multidisciplinary research and practical applications. To overcome the limitations of previous datasets and enhance measurement accuracy and consistency, all data within the ISSA were collected using spectrophotometers following a standardised measurement protocol, allowing objective comparisons across datasets. The variability using different measurement parameters such as measurement aperture size and pressure and the measurement repeatability was evaluated^24^ (see Technical Validation). Figure 1 provides a visual overview of the measurement instruments, the measuring process, the body locations, and the spectral reflectance across all skin measurements in the dataset.

The International Skin Spectra Archive (ISSA), comprises spectral data from 15,256 skin samples collected from 2,113 subjects. Each sample is meticulously documented in a single dataset record. These records detail the record number, origin, subject number, and skin type, including the subject’s ethnicity, gender, age, and body location. Additionally, they provide comprehensive instrument information such as type, inclusion of specular components, wavelength range, and interval. Alongside spectral data, the dataset also includes CIE colorimetric data for each sample. These measurements are based on the CIE 1931 standard colorimetric observer and the CIE standard illuminant D65 to simulate skin colour in daylight conditions. This illuminant provides a consistent and universally comparable lighting scenario that closely resembles average daylight, ensuring that our colorimetric assessments are both reliable and replicable across all samples.

**Fig. 2:**
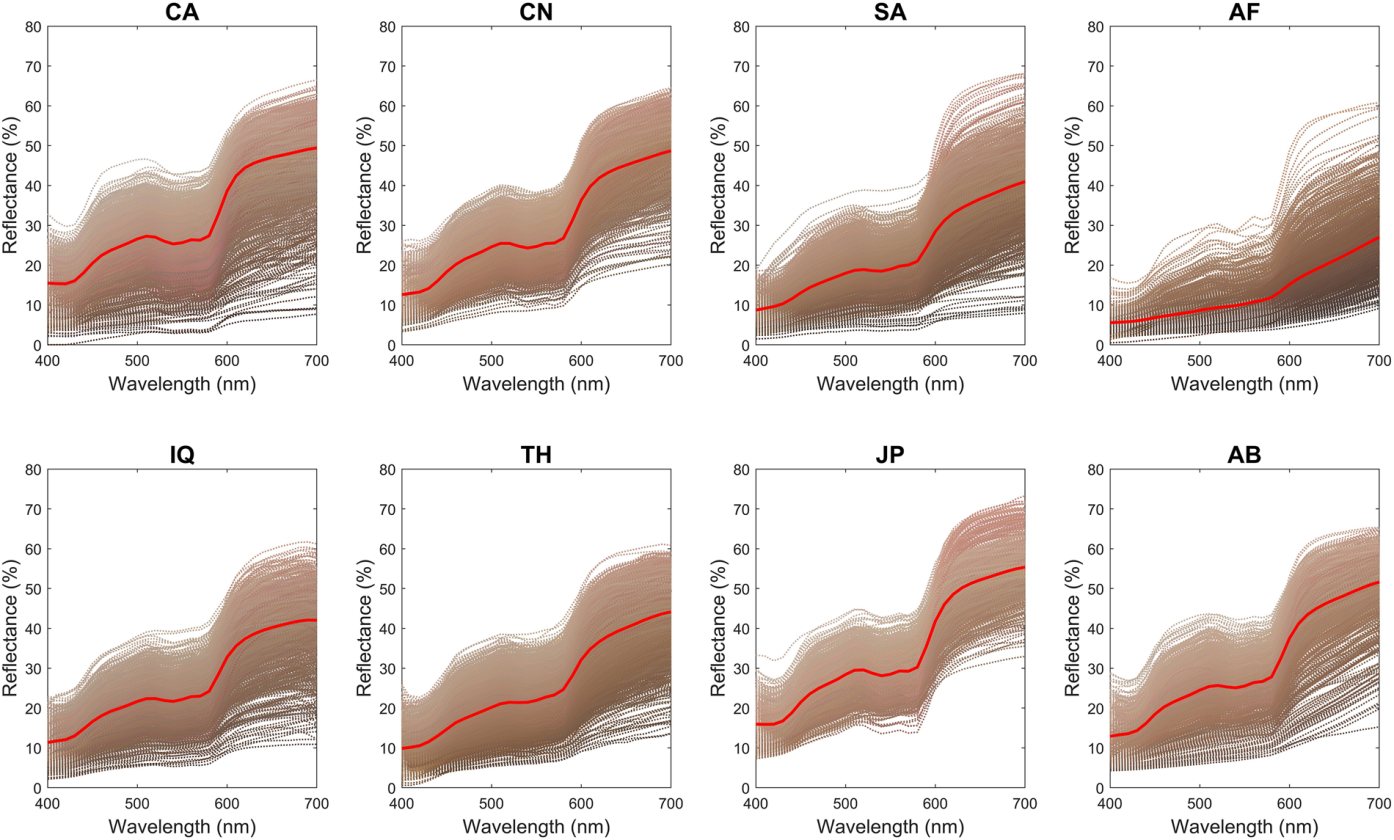
Spectral reflectance differences of human skin for the eight ethnic groups: CA (Caucasian), CN (Chinese), SA (South Asian - Pakistani), AF (African), IQ (Middle Eastern - Iraqi), TH (Southeast Asian - Thai), JP (Japanese), AB (Middle Eastern - Arabian). Each dotted line represents the spectral reflectance from a specific body location of an individual subject, with the colour of each line providing a visual approximation of the skin colour; the solid red line indicates the average spectral reflectance.

**Fig. 3:**
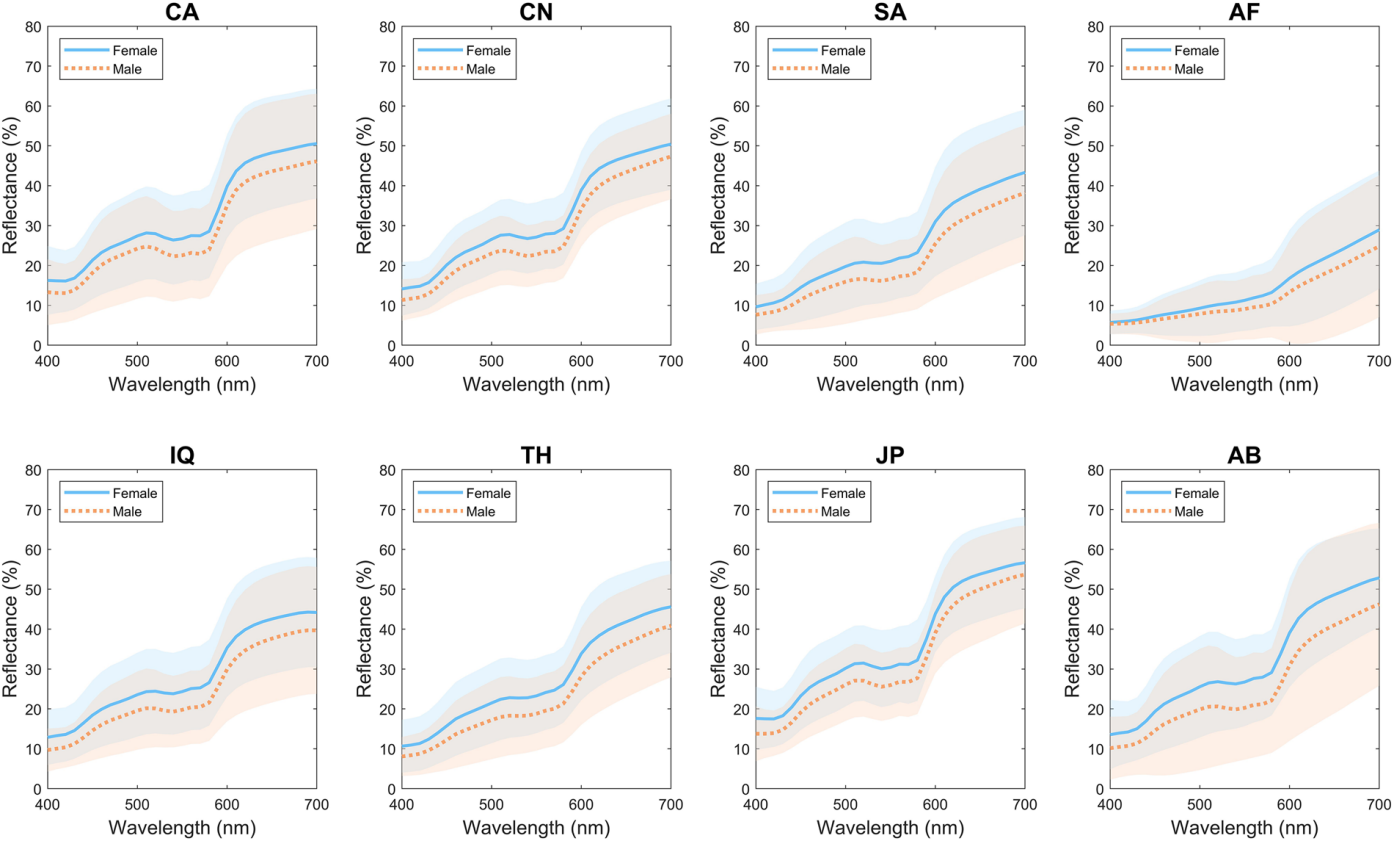
Spectral reflectance differences of female and male skin for the eight ethnic groups: CA (Caucasian), CN (Chinese), SA (South Asian - Pakistani), AF (African), IQ (Middle Eastern - Iraqi), TH (Southeast Asian - Thai), JP (Japanese), AB (Middle Eastern - Arabian). The solid lines represent the average spectral reflectance for female (blue line) and male (orange line), and the shaded areas denote ±2 standard error.

The spectral data from the ISSA offers extensive resources and references crucial for various applications, such as reflectance reconstruction, medical diagnosis, and training datasets for algorithms. Users can access specific groups of data tailored to particular needs, such as data of certain ethnic groups, genders, age groups, and body locations. Figures 2, 3 give examples to illustrate the varied human skin spectral reflectance across nationality or ethnicity and gender. These examples demonstrate the variability and specific patterns observable in the spectral data, emphasising the importance of considering these factors in skin colour analysis and research. The dataset also includes colorimetric data, enabling the direct assessment of skin colour appearance under standard D65 daylight illumination and CIE standard observer conditions. Additionally, colour appearance under different viewing conditions can be simulated using spectral data and any self-defined light sources and observer’s colour matching functions. The diversity and unique pattern highlighted in Fig. 1d illustrate the ISSA’s comprehensive coverage, providing valuable insights for research in skin physiology, genetics, and anthropology, and supporting innovation across a range of scientific and technological disciplines.

## Methods

### Data collection

Skin colour measurements were systematically obtained from thirteen global locations across eight countries as detailed in Table 1. The collection encompasses diverse ethnic groups across eleven datasets:

- Dataset 1: Comprises data from Caucasian subjects measured at the University of Sheffield and Manchester Metropolitan University in the United Kingdom.
- Dataset 2: Contains measurements of Chinese skin, conducted at institutions including the Beijing Institute of Technology, Minzu University of China, and Beijing Institute of Graphic Communication.
- Dataset 3: Includes measurements from Middle Eastern (Iraqi) individuals, undertaken at the University of Sulaimani in Iraq.
- Dataset 4: Southeast Asian (Thai) data gathered from Chulalongkorn University in Thailand.
- Dataset 5: South Asian (Pakistani) measurements taken at COMSATS University Islamabad in Pakistan.
- Dataset 6: Comprised of Caucasian samples, measured at University of Alicante in Spain.
- Dataset 7: A mixed collection involving Chinese, Caucasian, and South Asian subjects, conducted at University of Leeds in the United Kingdom.
- Dataset 8: African samples acquired at University of Leeds in the United Kingdom.
- Dataset 9: Japanese skin measurements obtained from Chiba University in Japan.
- Dataset 10: Chinese samples measured at the University of Science and Technology Liaoning in China.
- Dataset 11: Arabian skin measurements taken at Dar Al-Hekma University in Saudi Arabia.

**Table 1:** Description of the eleven sample datasets included in the ISSA: SP – Spectrophotometer.

Dataset	Year(s)	Location	Nationality or ethnicity Group(s)	Method(s)	Subjects	Body Locations
1	2012–2014	UK	Caucasian	SP	172	9
2	2013	China	Chinese	SP	201	9
3	2013	Iraq	Middle Eastern (Iraqi)	SP	149	9
4	2014	Thailand	Southeast Asian (Thai)	SP	710	6
5	2015	Pakistan	South Asian (Pakistani)	SP	117	10
6	2016	Spain	Caucasian	SP	27	3
7	2017–2018	UK	Chinese, Caucasian, South Asian	SP	249^a^	4
8	2019–2023	UK	African	SP	147	4–10
9	2023–2024	Japan	Japanese	SP	118	9
10	2024	China	Chinese	SP	116	7
11	2024	Saudi Arabia	Middle Eastern (Arabian)	SP	107	9

^a^including 155 Caucasian, 53 Chinese, and 41 South Asian.

Ethical approval for this study was obtained from the following committees: the University Research Ethics Committee (School of Clinical Dentistry), The University of Sheffield (Reference Number: 9); the IPHS Research Ethics Committee, University of Liverpool (Reference Number: IPHS-1314-278); the PVAC & Arts joint Faculty Research Ethics Committee, University of Leeds (Reference Number: PVAR 13-057) and the Faculty of Arts, Humanities and Cultures Research Ethics Committee, University of Leeds (Reference Number: LTDESN-067). Additional datasets were collected under the ratification of host institutions, which accepted the ethical approval from the University of Sheffield, Liverpool or Leeds without requiring additional approvals. For Dataset 4 (Chulalongkorn University, Thailand), the study complied with the university’s policy in 2014, which exempted research that did not link data to individual participants. All other datasets were approved by their respective institutional ethics committees, including the Ethics Committee of the University of Science & Technology Liaoning and the Research Ethics Committee of the Scientific Research Centre at Dar Al-Hekma University. All participants across all collection sites provided written informed consent, which included permission to share anonymised data for research purposes.

Note that subjects under age 18 were excluded from the data collection; only healthy skin without visible abnormalities was included in the dataset. Despite its comprehensive scope, the current dataset does not fully represent all segments of the global population. For example, it lacks measurements from Africans living in Africa and individuals of mixed ethnicity. We acknowledge these limitations and intend to collect more data to update this dataset further, aiming to address these gaps and enhance its inclusivity and representativeness.

### Principles of skin colour measurement

For over 70 years, CIE colorimetry has been successfully applied for the objective measurement of colour^34^. The principles of skin colour measurement in this study adhere strictly to the CIE colourimetry standards. The CIE system of colorimetry is widely used for the calculation of appropriate colour-related parameters, for example CIELAB coordinates, from spectral reflectance measurements of surfaces using spectrophotometers with the assumption of a CIE standard observer, and under standard illuminant (e.g., D65, which represents average daylight with a correlated colour temperature of 6504 K)^35^. This standard illuminant is crucial as it provides a consistent basis for colour evaluation, mimicking natural light to ensure that colour measurements are comparable irrespective of the actual lighting conditions under which they are taken. Both skin colour appearance and skin colour differences can be further predicted in the CIELAB uniform colour space. Although CIELAB coordinates are extensively used in industry to assess the ‘colour’ of a sample, they are typically computed under the standard daylight illuminant and, once calculated it is not easy to re-calculate values for a second, different illuminant^36^. To accommodate applications that require assessments under multiple lighting conditions, the ISSA provides spectral data which can be used to calculate appropriate colorimetric coordinates under any illuminant as long as it is defined by a relative spectral power distribution, either tabulated, as in the case of CIE standard illuminants or measured with an appropriate instrument. This flexibility is crucial not only for accurately simulating skin appearance under different lighting and measuring colour discrimination thresholds but also for extracting detailed information about skin characteristics and chromophores, which is important for various technological and medical applications.

### Measurement instruments

Portable spectrophotometers (SP) were employed for all skin spectral reflectance measurements. An SPM measures the spectral reflectance factor, defined as the ratio of the radiant flux reflected by a sample to that of a perfect diffuser, normally a calibrated white ceramic tile. Each instrument incorporates a diffused light source, ensuring that measurements remain consistent across conditions. This method is independent of the spectral power distribution and does not account for fluorescence effects. The SPM’s aperture directly contacts the sample, eliminating ambient light interference, as demonstrated in Fig. 1b where a portable spectrophotometer is used on a subject’s forehead. Also, the instrument uses a pulsed xenon lamp with a UV cut-off filter such that the measurement time is extremely short at approximately 1 second. This should serve to minimise the possible effects of movement during the measurement period bearing in mind that the instrument is being held at the required location on the subject’s body. The instrument is also able to include or exclude the specular component of the reflected light. In this dataset, the specular component of the light reflected from the sample was included in the measurements.

In this study, Datasets 1, 2, 3, 6, and 10 were measured with a Konica Minolta CM-2600d (Tokyo, Japan). Datasets 5, 7, 8, 9, and 11 were measured with a Konica Minolta CM-700d (Tokyo, Japan). Dataset 4 was measured with an X-Rite SP62 (Michigan, US). All the devices adhered to the CIE di:8° geometry standards with diffuse illumination and an 8-degree detection angle^34^. Measurements were taken in the specular component included (SCI) mode with an aperture size of either 3 mm or 8 mm. Most skin reflectance data span the visible spectral range from 360 nm to 740 nm, although some datasets are confined to a narrower range of 400 nm to 700 nm, all recorded at 10 nm intervals.

### Measurement protocol

The nationality or ethnicity, gender, age, and body location of the subject are important and depend on the requirements of the specific study that needs the measurement of human skin colour. Thus, this information for each participant was first determined through self-evaluation via a questionnaire. In regions with homogeneous populations (e.g., China, Thailand, Japan), all participants belonged to the same ethnic group. In regions with mixed populations (e.g., the UK), participants were provided with a questionnaire that included options for existing ethnicities, mixed ethnicity, and “other” (self-defined). This information was recorded using consistent coding schemes in the dataset (see Data Records).

To ensure the integrity and consistency of all skin spectral reflectance measurements in this study, several standardised conditions were rigorously maintained. First, it was essential for the skin of all subjects to be clean, unabraded, and free from any cosmetics, lotions, or medical products that could affect the measurement outcomes. Each subject was prepared accordingly prior to data collection to meet this standard. Additionally, the measurement instruments (portable spectrophotometer - SP) were calibrated according to manufacturer guidelines before each session. Lighting conditions for measurements were carefully controlled; all measurements were conducted under diffuse lighting conditions to avoid discrepancies associated with collimated light sources. This was facilitated by lighting systems integrated within the measurement instruments (SP). Such controlled environments guaranteed that the spectral data collected was accurate and consistent across all subjects and datasets.

During the measurement, the portable spectrophotometer was brought to the subject, ensuring the sample area had no blemishes (e.g. hair, freckles, etc.) and had not been subjected to recent pressure (e.g. to promote blood flow). Areas with visible hair (e.g., the chin of male participants with beards) were avoided during measurements to ensure that only clear skin areas were included in the dataset. When using a spectrophotometer, care was taken to ensure the instrument was gently in contact with the area of skin to be measured, to prevent extraneous light from reaching the detector (see Fig. 1b). It was also necessary to be careful that no excessive pressure was applied to the skin when contacting the device, which might lead to a change in its colour due to the promotion of blood flow beneath the surface. Measurement parameters mentioned in the last section, such as the geometry of illumination and the inclusion of the specular component, must be checked before measurement. Measurements were taken at 4–10 different body locations depending on the site, with the forehead, cheek, and back of the hand covered by all sites. For consistency, measurements were taken from one randomly selected side (left or right) of each subject. Laterality was generally not recorded, except for Datasets 7 and 8, which included bilateral cheek measurements.

After completing the measurements, we reviewed the data and excluded any measurements with zero reflectance (e.g., due to instrument error) or any participant data with colour difference greater than 15 $\triangle {E}_{{\rm{ab}}}^{* }$ between positions. All measurement data were then carefully recorded and reported in accordance with the predefined coding schemes. This protocol not only supports the reliability of our measurements but also enhances the comparability of our data across various locations and time periods.

### Computing colorimetric values

Based on the CIE colourimetry, the skin spectral data in our study are transformed into CIE colorimetric data using the CIE standard colorimetric observer and CIE standard illuminant D65, the latter simulating the skin colour under daylight conditions. These derived colorimetric data include tristimulus values: *X*, *Y*, *Z*; x, y chromaticity coordinates: *x*, *y*; CIELAB parameters: L*, a*, b*, ${C}_{{\rm{ab}}}^{* }$, *h*_ab_; u’, v’ chromaticity coordinates: *u*′, *v*′, respectively^35^.

The tristimulus values *X*, *Y*, *Z* are obtained:

$${\rm{X}}={\rm{k}}\sum _{{\rm{\lambda }}}S\left({\rm{\lambda }}\right){\rm{R}}({\rm{\lambda }})\bar{{\rm{x}}}({\rm{\lambda }})\triangle {\rm{\lambda }}$$

$${\rm{Y}}={\rm{k}}\sum _{{\rm{\lambda }}}S\left({\rm{\lambda }}\right){\rm{R}}({\rm{\lambda }})\bar{{\rm{y}}}({\rm{\lambda }})\triangle {\rm{\lambda }}$$

$${\rm{Z}}={\rm{k}}\sum _{{\rm{\lambda }}}S\left({\rm{\lambda }}\right){\rm{R}}({\rm{\lambda }})\bar{{\rm{z}}}({\rm{\lambda }})\triangle {\rm{\lambda }}$$

where *S*(*λ*) is the spectral power distribution (SPD) of the CIE standard illumination D65; $${\rm{R}}($$*λ*$$)$$ is the spectral reflectance of the skin sample as a function of wavelengths; $$\bar{{\rm{x}}}$$(λ),$$\,\bar{{\rm{y}}}$$(λ), and $$\bar{{\rm{z}}}$$(λ) are colour-matching functions of the CIE standard observer; λ is the wavelength (in the unit of nm); k is a scaling constant to normalise the tristimulus values.

CIE chromaticity coordinates, x, y, z, are defined as the equations below:

$${\rm{x}}=\frac{X}{X+Y+Z};\,{\rm{Y}}=\frac{Y}{X+Y+Z};\,{\rm{Z}}=\frac{Z}{X+Y+Z}$$

where x + y + z = 1.

The coordinates of CIELAB are transformed from the XYZ tristimulus values:

$${\rm{L}}* =116f({\rm{Y}}/{\rm{Yn}})-16$$

$${\rm{a}}* =500[f({\rm{X}}/{\rm{Xn}})-f({\rm{Y}}/{\rm{Yn}})]$$

$${\rm{b}}* =200[f({\rm{Y}}/{\rm{Yn}})-f({\rm{Z}}/{\rm{Zn}})]$$

$$f\left(w\right)=\left\{\begin{array}{c}{(w)}^{1/3}\,{for}\,{w} > 0.008856\\ 7.787\left(w\right)+16/116\,{for}\,{w}\le 0.008856\end{array}\right.$$

where X, Y, and Z and Xn, Yn, and Zn represent the tristimulus values of the object colour and reference white, respectively. And the perceived attributes of chroma ${{\rm{C}}}_{{ab}}^{* }$ and hue h_*ab*_ can be predicted in CIELAB colour space:

$${{\rm{C}}}_{{ab}}^{* }={[({{a}^{* })}^{2}+({{b}^{* })}^{2}]}^{1/2}$$

$${{\rm{h}}}_{{ab}}={\tan }^{-1}({b}^{* }/{a}^{* })$$

The u’, v’ chromaticity coordinates are transformed from the XYZ tristimulus values:

$${\rm{u}}{\prime} =4{\rm{X}}/({\rm{X}}+15{\rm{Y}}+3{\rm{Z}})$$

$${\rm{v}}{\prime} =9{\rm{Y}}/({\rm{X}}+15{\rm{Y}}+3{\rm{Z}})$$

This computing process is seamlessly integrated into the dataset file, ensuring that the colorimetric values are directly accessible and systematically calculated to reflect accurate and consistent colour assessments under standardised daylight conditions.

## Data Records

**Fig. 4:**
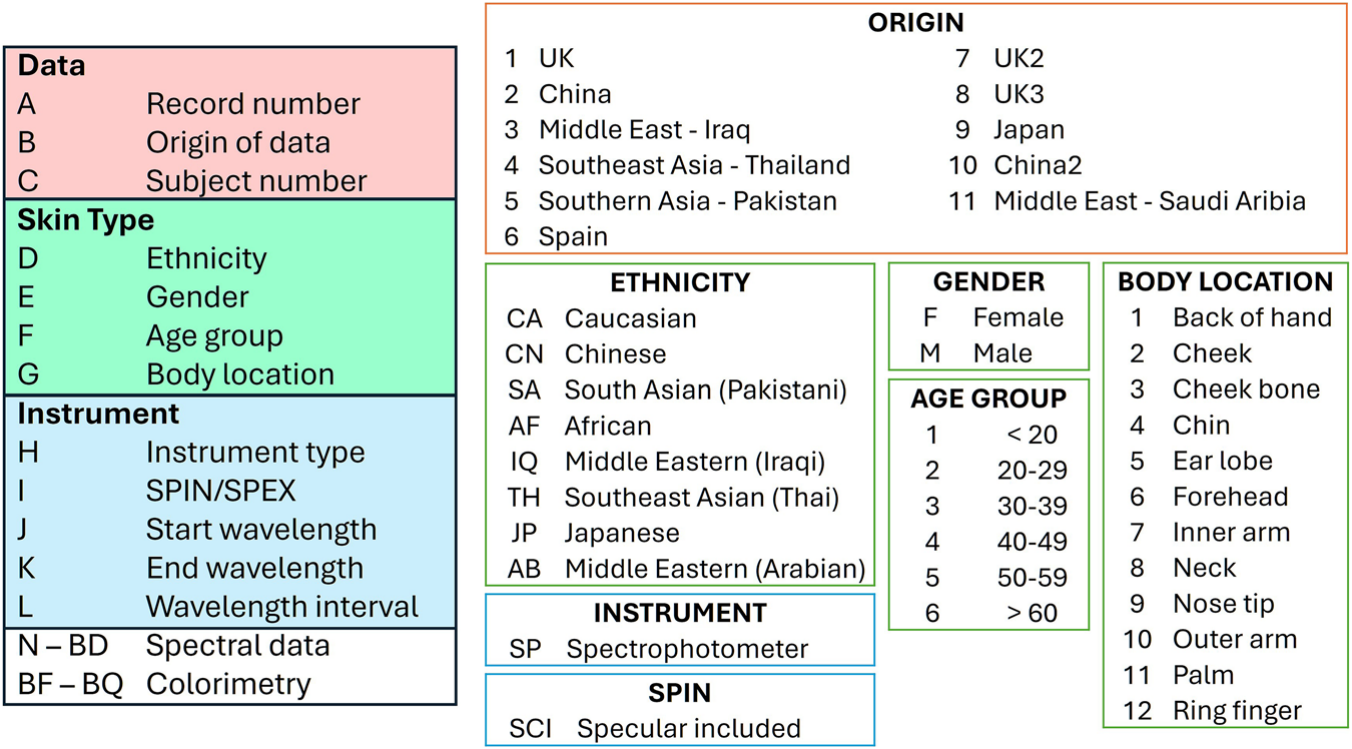
Overview of data structure and coding scheme.

The International Skin Spectra Archive (ISSA) has been deposited at *figshare*^37^, specifically structured for ease of use in coding applications. The file is organised into two spreadsheets: one coding scheme and one datasheet. Figure 4 shows the overview of the data structure and coding schemes. Within the datasheet, data are arranged into columns labelled A to BQ, each representing specific attributes of the recorded measurements. A is the unique identifier for each record; B is the origin of data, linked to the detailed origin table in the coding scheme spreadsheet (also see the top right column in Fig. 4); C is the unique identifier for each subject; D is the ethnicity, linked to the ethnicity key in the coding scheme (also see the middle right column in Fig. 4); E is the gender; F is the age group linked to the age group categories; G is body location, linked to the body location descriptions; H is the instrument type; I is the specular components mode; J and K are the start and end wavelength measured in nanometres (nm); L is the wavelength interval of measurement in nm. The spectral data are given in columns N (360 nm) to BD (780 nm). All records include at least the spectral range of 400–700 nm, which covers the full visible spectrum. Some datasets extend beyond this range, capturing additional data from 360–740 nm. CIE colorimetric data, calculated using the CIE standard colorimetric observer and CIE standard illuminant D65, are listed in columns BF to BQ.

## Technical Validation

The dynamic nature of skin colour and its variability due to many factors as mentioned make consistent measurement difficult, especially in comparison to flat uniform samples. To address this, our methodology involves not only a stringent protocol that ensures consistent measurements across different laboratories and instruments but also a thorough assessment of the variability of skin measurements, focusing on the short-term repeatability and the inter-instrument agreement. The short-term repeatability is evaluated by repeating the measurement of the same body location, using the same instrument in quick succession, ensuring consistent pressure on the skin. Inter-instrument agreement is assessed by measuring the same body location using different settings on the same instrument. The CIELAB colour difference is used to quantify the variability between the measurements^34^. Our results indicate that the short-term repeatability for the SP maintains a variation within 0.40 $\triangle {E}_{{\rm{ab}}}^{* }$ across different pressure and aperture sizes. The mean colour differences between the different values of measurement pressure were 0.88 and 1.82 for the medium and small aperture respectively. The mean colour differences between the two measurement apertures were 2.48 and 3.04 for the two different pressures, respectively. The greater effect of field size on the variability, compared with that of pressure, implies that the variability in the colour distribution of the area being measured is greater than the variability in the measurement caused by the pressure: thus reasonable pressure on the skin does not significantly affect the measured colour values. Those variances on live human skin are higher than those observed with uniform flat skin-colour samples, such as the PANTONE SkinTone™ Guide. A detailed investigation of the variability in skin colour measurements, as explored in a prior study^24^, underpins these findings and contributes to our understanding of the complexities involved in skin colour measurement. The validation results have provided useful guidance for the definition of measurement protocol and the establishment of the current skin spectral dataset.

## Usage Notes

The dataset is designed for easy integration into coding environments, such as R Studio or MATLAB. The spreadsheet is formatted to include the code necessary for calculating the CIE colorimetric data using the CIE standard colorimetric observer and CIE standard illuminant D65. Additionally, users have the flexibility to use spectral reflectance data to compute colorimetric values based on any self-defined illuminants and observer’s colour matching functions, facilitating versatile applications across different research and practical contexts.

## Data Availability

No custom code was used to generate the data described in this study.
